# Radiation-induced DNA damage and repair in human γδ and αβ T-lymphocytes analysed by the alkaline comet assay

**DOI:** 10.1186/2041-9414-1-8

**Published:** 2010-06-08

**Authors:** Halina Lisowska, Marta Deperas-Kaminska, Siamak Haghdoost, Ingela Parmryd, Andrzej Wojcik

**Affiliations:** 1 Jan Kochanowski University, Department of Radiobiology and Immunology, Kielce, Poland; 2 Joint Institute for Nuclear Research, Dubna, Russia; 3 Department of Genetics Microbiology and Toxicology, Stockholm University, Sweden; 4 The Wenner-Gren Institute, Stockholm University, Sweden

## Abstract

It has been shown by a number of authors that the radiosensitivity of peripheral blood mononuclear cells (PBMC) is higher in cancer patients compared to healthy donors, which is interpreted as a sign of genomic instability. PBMC are composed of different cell subpopulations which are differently radiosensitive and the difference between cancer patients and healthy donors could also be due to different composition of their PBMC pools. Gamma-delta T-lymphocytes play an important role in immunosurveillance and are promising cells for immunotherapy. Their abundance is frequently reduced in cancer patients so should their sensitivity to radiation be lower than that of other T-lymphocytes, this could, at least partly explain the low radiosensitivity of PBMC from healthy individuals compared to cancer patients. The present investigation was carried out to test this. Using the alkaline comet assay we analysed the level of DNA damage and repair in isolated γδ T-lymphocytes, pan T-lymphocytes and in total PBMC exposed *in vitro *to gamma radiation. We found no difference in the level of DNA damage and the capacity of DNA repair between the T cell populations. This is the first study that addresses the question of sensitivity to radiation of gamma-delta T-cells.

## Background

Human peripheral blood mononuclear cells (PBMC) are used as surrogate tissue for the assessment of individual sensitivity to ionising radiation [1]. Using the G_2 _chromosomal aberration assay it has been shown by a number of authors that the radiosensitivity of PBMC is higher in cancer patients compared to healthy donors [2-9]. A similar result was reported using the micronucleus assay [10] and the comet assay [11,12] and is generally interpreted as a reflection of genomic instability in PBMC of cancer patients [10].

PBMC are composed of different cell subpopulations which are differently radiosensitive. It is generally accepted that B lymphocytes show a higher radiosensitivity than T-lymphocytes that are all CD3+ [13-16]. Among the T-lymphocytes cytotoxic CD8+ cells appear somewhat more sensitive than helper CD4+ cells [16,17], although this was not observed in all studies [18]. A lymphocyte subpopulation which recently attracted interest is composed of T-lymphocytes with γδ T-cell receptors (TCR). The reason for this is the discovery that these cells contribute to immunity against cancer [19]. In peripheral blood γδ lymphocytes account for less than 5% of total T-lymphocytes while their proportion in the intestine can be much higher [20].

Given their role in immunosurveillance, the level and/or the activity of γδ T-lymphocytes is expected to be low in PBMC of cancer-prone individuals and high in PBMC of individuals who are resistant to cancer. This is supported by findings in patients with lymphoma, myeloma, breast and nasopharyngeal carcinoma [21-23]. Should the sensitivity to radiation of γδ lymphocytes be lower than that of the more common αβ T-lymphocytes, this could, at least partly explain the low radiosensitivity of PBMC from healthy individuals compared to cancer patients. The present investigation was carried out to test if there are differences in the radiation sensitivity between different TCR subtype populations by comparing the level of DNA damage and the kinetics of DNA repair in isolated γδ T-lymphocytes, pan T-lymphocytes and in total PBMC exposed *in vitro *to gamma radiation. Experiments were performed using the alkaline comet assay. Cells were exposed to the moderate dose of 1 Gy at 37°C.

## Results

**Figure 1:**
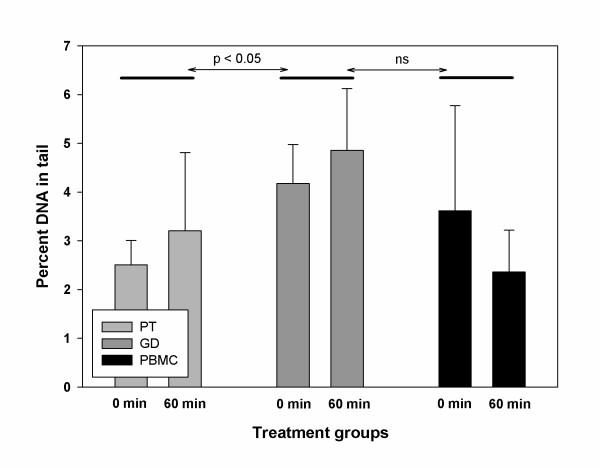
**Spontaneous levels of DNA damage measured after 0 min and 60 min of incubation time**. Error bars: standard deviations from the mean % TDNA values. PT: pan T, GD: gamma-delta, PBMC: peripheral blood mononuclear cells, ns: non significant.

We decided to analyse the percentage of DNA in the tail (% TDNA) as marker of damage [24]. The control % TDNA values did not differ significantly between 0 and 60 min of incubation at 37°C (Figure 1). The average level of spontaneous damage was highest in γδ T-lymphocytes and it was significantly higher than in pan T-lymphocytes. The differences between γδ T-lymphocytes and PBMC, as well as between pan T-lymphocytes and PBMC were not significant.

**Figure 2:**
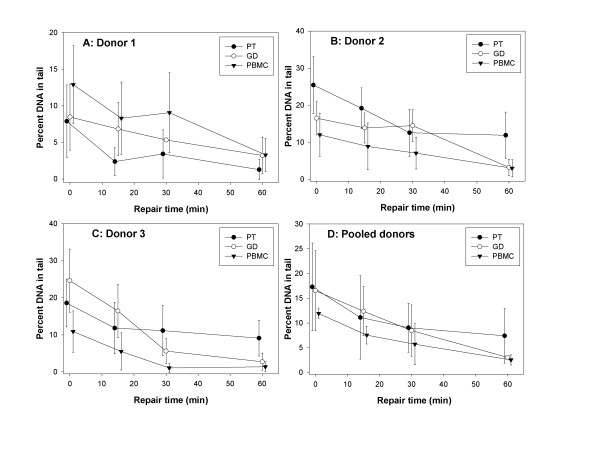
**Kinetics of DNA repair in cells from three independent experiments each with with lymphocytes of one donor donor (panels A-C) and pooled results (panel D)**. Net percent of DNA in tail values are shown. Error bars: standard deviations from individual cell measurements (panels A-C) and from the mean % TDNA values (panel D). PT: pan T, GD: gamma-delta, PBMC: peripheral blood mononuclear cells.

The % TDNA values found in the control cells were subtracted from the radiation-induced values. The net radiation-induced % TDNA values as a function of repair time are shown in figure 2. No consistent difference between the cell subpopulations was observed for the initial level of DNA damage. DNA repair proceeded in all cell types at a similar rate and following 60 min of repair the majority of DNA damage was repaired. No consistent difference between the cell subpopulations was observed for the residual level damage.

**Figure 3:**
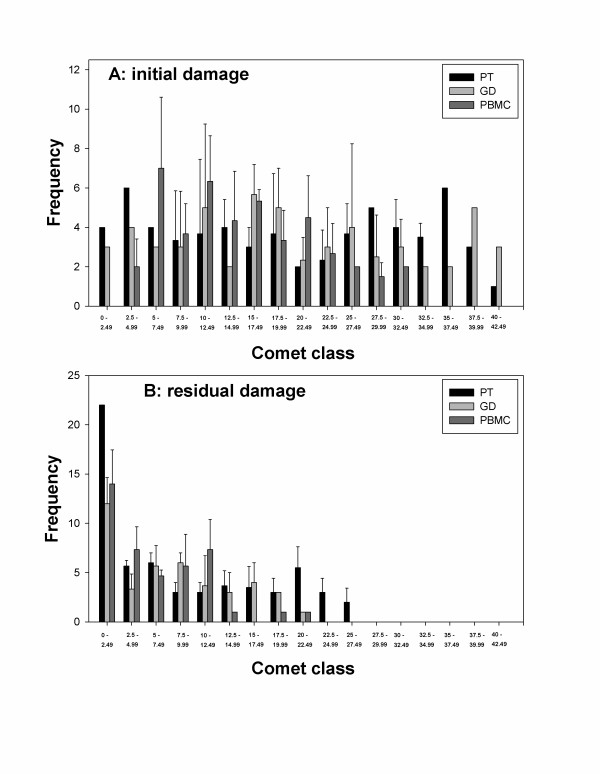
**Distributions of comets scored after 0 min repair (initial damage - panel A) and 60 min repair (residual damage - panel B)**. Cells were grouped into classes of percent of DNA in tail values. Pooled results from three experiments. Error bars: standard deviations from independent experiments. PT: pan T, GD: gamma-delta, PBMC: peripheral blood mononuclear cells.

An advantage of the comet assay is the ability to analyse DNA damage in individual cells. Although we observed no difference in the mean level of DNA damage between the analysed cell subpopulations, it is possible that differences existed in the distribution patterns of cells with different level of DNA damage. In order to check this, cells were grouped into classes of % TDNA values and plotted on one graph. The results for 0 and 60 min repair are shown in figure 3. The distribution patterns of all three cell populations look similar.

## Discussion

It has been proposed that the *in vitro *radiosensitivity of PBMC correlates with individual sensitivity to ionising radiation [1] and it has been shown that the *in vitro *radiosensitivity of PBMC is higher in cancer patients compared to healthy donors [2-12]. The mechanisms behind these observations are not known. One factor could be a different composition of lymphocyte subsets in PBMC collected for analysis from radiosensitive and radioresistant donors.

Differences in the radiosensitivity of lymphocyte subsets have been demonstrated, although the results are contradictory. A high sensitivity of B-, as compared to T-lymphocytes has generally been found (see [14,16] and the literature within), whereas one study found no difference between the subsets after high doses of gamma radiation and after exposure of cells to neutrons [15]. Both Wilkins et al. [17] and Schmitz et al. [16] observed a somewhat higher sensitivity in CD8+ than in CD4+ T-lymphocytes. No difference between CD8+ and CD4+ T-lymphocytes was observed by Louagie at al. [18], who however, found that both CD8+ and CD4+ T-cells were more radiosensitive than NK cells. In these studies, αβ and γδ T-cells were not separated meaning that the bulk of both CD8+ and CD4+ T-lymphocytes would have had the αβ receptor. We have, for the first time, compared the radiosensitivity of γδ T-lymphocytes with that of pan T-lymphocytes and PBMC. Pan T-lymphocytes are composed of both αβ and γδ T-lymphocytes, however, the fraction of the latter in lymphocytes of our donors was not higher than 3% (not shown), so their influence on the radiosensitivity of pan T-lymphocytes is negligible. We found no difference in the level of radiation-induced DNA damage and the capacity of DNA repair between the cell populations.

Our result shows that the low radiosensitivity of PBMC isolated from healthy donors compared to cancer patients, can not be due to a high level of γδ T-lymphocytes in the peripheral blood of the former. This does not exclude the possibility that the difference in radiosensitivity results from a different composition of lymphocyte subpopulations in healthy and cancer patients. However, this possibility has hitherto not been studied.

An interesting finding was that the level of spontaneous DNA damage was higher in γδ T-lymphocytes than in pan T-lymphocytes and in PBMC. This finding is somewhat difficult to interpret. The essential factor determining whether a segment of DNA appears in the tail rather than in the head of a comet is the relaxation of DNA supercoiling which is a consequence of DNA damage [24]. This would suggest that γδ T-lymphocytes suffer from a high level of spontaneous DNA damage. The reason for this is unclear and in our opinion the result should be treated with caution. Obviously, it must be validated by more studies.

An observation that requires a comment is the large scatter of the comet results as seen in figure 2. We scored the slides in a blind manner and the large standard deviations are due to the presence of both intact and damaged cells on the same slide. We tried to reduce the scatter by eliminating from analysis an equal number of cells with lowest and highest level of damage, but the standard deviations remained broad. A lower scatter would possibly be found if we used a higher dose of radiation than 1 Gy. This would lead to a higher level of DNA damage. However, we intentionally kept the dose at 1 Gy, because of the known high radiosensitivity of some lymphocyte subsets [25]. In our opinion a dose higher than 1 Gy could have damaged the lymphocytes to an extent that would make the results unreliable.

It should be stressed that despite our negative results related to the sensitivity of cells to radiation, γδ T-lymphocytes, in particular those having the γ9δ2 TCR, are interesting cells to exploit for immunotherapy because they specifically cause lysis of tumour cells without affecting non transformed cells [26] and clinical trials show promising results with γδ T lymphocyte immunotherapy [27]. The combination of immunotherapy with *in vitro *expanded γδ T-lymphocytes and radiotherapy has not been evaluated and is an avenue worth visiting.

## Conclusions

Taken together, the results provide no evidence for different radiation sensitivity of γδ lymphocytes, pan T-lymphocytes and total PBMC. This does not exclude the possibility that the often observed difference in radiosensitivity of PBMC between cancer patients and healthy donors results from a different composition of lymphocyte subpopulations.

## Methods

### Isolation of human peripheral mononuclear cells and T-lymphocytes

Experiments were performed with PBMC isolated from three healthy volunteers: donor 1 (male, aged 49, non-smoker), donor 2 (male, aged 53, non-smoker) and donor 3 (male, aged 40, non-smoker). The study was approved by the local ethical committee. Written informed consent was obtained from the volunteers for publication. A copy of the written consent is available for review by the Editor-in-Chief of this journal. Three independent experiments were performed, each with blood of one donor.

PBMCs were isolated from 80 ml fresh blood on a histopaque gradient. Pan T cells (CD3+, containing both αβ and γδ T-lymphocytes) were isolated from 6 × 10^6 ^PBMCs that were mixed with pan T-cell isolation beads according to the manufacturers instructions (Miltenyi Biotec Inc., Auburn, CA). T-lymphocytes being CD3+ were negatively selected on a MACS column. 4 × 10^6 ^PBMCs were kept for further experiments and pan γδ T-cells were purified from the remainder of the PBMCs assuming that 10% were γδ T-cells and adjusting the manufacturers protocol accordingly (Miltenyi Biotec Inc., Auburn, CA). γδ+ cells were positively selected on a MACS column. The cells were counted in trypan blue in a Countess Automatic Cell Counter (Intvitrogen, Carlsbad, CA) and kept at 1 × 10^6 ^per ml in RPMI medium supplemented with 2 mM L-glutamine, 100 U/ml penicillin and 100 mg/ml streptomycin at 37°C in humidified incubator under 5% CO_2 _and used within twenty hours for the comet assay.

### Irradiation and comet assay

Irradiation was carried out using a Scanditronix (Uppsala, Sweden) ^137^Cs source operating at 0.47 Gy/min. Cells were transferred to 1.5 ml Eppendorf cups, placed in 37°C water cups, irradiated with a dose of 1 Gy and immediately placed on ice to stop DNA repair. Following incubation on ice cells were kept at 37°C for 0, 15, 30 and 60 min and processed for the alkaline comet assay [28]. Two non-irradiated samples per cell type and experiment were analysed as controls following incubation at 37°C for 0 and 60 min.

Microscope slides were pre-coated with 50 μl of 0.5% normal melting agarose (BDH, Germany), and left to dry. 1.5 × 10^5 ^cells were mixed 1:1 with low melting agarose (US, Belgium) at 37°C, 100 μl of this suspension was placed on a pre-coated slide, covered with a coverslip and left to solidify at +4°C for 5 min. Thereafter the coverslips were removed and the rest of the assay was conducted at +4°C, to ensure gel stability, using only red light as the light source. The slides were immersed in lysis buffer (2.5 M NaCl, 100 mM Na_2_EDTA, 10 mM Tris, 1% Triton X-100, pH 10.0) for 1 hour, followed by a brief washing step in double distilled H_2_O for 5 min. The slides were then placed in an EC 340 Maxicell Primo (Thermo EC, Holbrook, USA) electrophoresis tank containing 1.31 L unwinding/electrophoresis buffer (1 mM Na_2_EDTA, 300 mM NaOH, pH 13.3). After 1 h of incubation the power supply unit (Kyoritsu P 103B, Tokyo, Japan) was switched on and ran at 32.5 V (1 V/cm), 430 mA for 25 minutes. The number of slides in the unit was constant for all experiments.

Following electrophoresis, slides were washed in neutralization buffer (0.4 M Tris), stained with 1 μM DAPI (in water, Sigma-Aldrich), covered with coverslips and stored in the dark at +4°C in a humid container until analysis the following day. Comets were scored blind using a 40× objective on a Nikon Eclipse E800 (Nikon, Tokyo, Japan) fluorescence microscope. Images were acquired and analyzed using the Comet II software (Perspective Instruments, Suffolk, UK, version 2.11). 50 cells per experiment and point were analysed.

### Statistical analysis

The Comet II software yields Excel data sheets with the measurement results for each cell from a treatment group. We focused on analysing % TDNA as the marker of damage. In order to reduce data scatter, the results obtained for each treatment point were processed as follows: the % TDNA values were sorted in raising order and 5 lowest and 5 highest values were deleted. This procedure was carried out for every treatment group including the control samples. Thus, the number of cells analysed was reduced to 40. For the analysis of radiation-induced level of % TDBA, the mean (from 0 and 60 min incubation time) control level of % TDNA was subtracted.

The average values from individual experiments were compared using the 2 tailed Student's t-test.

## Competing interests

The authors declare that they have no competing interests.

## Authors' contributions

AH and MD-K ran the comet assay experiments; IP isolated lymphocytes, participated in the design of the study and in writing the manuscript; SH collected peripheral blood and participated in writing the manuscript; AW participated in the design of the study, in statistical analysis and in writing the manuscript. All authors read and approved the final manuscript.
